# Using illicit drugs alone in Vancouver, Canada: a gender-based analysis

**DOI:** 10.1186/s13011-025-00637-x

**Published:** 2025-02-22

**Authors:** Kat Gallant, Kanna Hayashi, Eric C. Sayre, JinCheol Choi, Manal Mansoor, Thomas Kerr

**Affiliations:** 1 British Columbia Centre On Substance Use, Vancouver, BC Canada; 2 Interdisciplinary Studies Graduate Program, University of British Columbia, Vancouver, BC Canada; 3 Faculty of Health Sciences, Simon Fraser University, Vancouver, BC Canada; 4 Department of Medicine, University of British Columbia, Vancouver, BC Canada

**Keywords:** Substance Use, Using Alone, Gender, People Who Use Drugs, Solitary Drug Use

## Abstract

**Objectives:**

Canada continues to experience an epidemic of toxic drug-related overdose deaths. Public health messaging emphasizes the dangers of using drugs alone as it restricts timely overdose response or renders it impossible, yet this practice remains prevalent among people who use drugs. While drug use practices and associated harms are known to be highly gendered, little is known about how factors shaping solitary drug use may differ across genders (including cisgender men, cisgender women, transgender women, Two-Spirit people and gender diverse people). Thus, we sought to explore solitary drug use practices according to gender in Vancouver, Canada.

**Methods:**

Data were collected through Vancouver Injection Drug Users Study, a prospective cohort study between June 2019 and May 2023. We used gender-stratified multivariable generalized estimating equation models to identify factors associated with using drugs alone.

**Results:**

Among the 697 participants, 297 (42.6%) reported using drugs alone in the previous 6 months at baseline. In multivariable analyses, we found that being in a relationship was negatively associated with using alone for both cisgender men and cisgender women (adjusted odds ratio [AOR] = 0.25 and 0.34, respectively), while homelessness was negatively associated for cisgender men only (AOR = 0.45). Factors positively associated for cisgender men included daily illicit stimulant use (AOR = 1.90), and binge drug use (AOR = 2.18). For cisgender women, only depression was positively associated with using drugs alone (AOR = 2.16). All *p*-values < 0.05. While unable to conduct a multivariable analysis on transgender, Two-Spirit and gender diverse people due to small sample sizes, bivariate analyses showed larger impact of depression on using alone for Two-Spirit (OR = 8.00) and gender diverse people (OR = 5.05) compared to others, and only gender diverse people’s risk was impacted by experiences of violence (OR = 9.63). All *p*-values < 0.05.

**Conclusion:**

The findings of this study suggest significant heterogeneity in gender-specific factors associated with using drugs alone. Factors exclusively impacting cisgender men’s risk included homelessness and daily stimulant use, and depression having a significant impact on cisgender women’s, but not cisgender men’s, risk. Ultimately, gender-specific factors must be recognized in public health messaging, and in developing policies and harm reduction measures to address the risks associated with using alone.

## Background

Canada continues to experience an epidemic of drug-related overdose deaths, with an average of 22 deaths daily in 2023, and one-third occurring in the province of British Columbia (BC) [63]. In spite of recent province-wide initiatives, including expanding access to opioid agonist treatment options and take-home naloxone programs [52, 54], overdose rates have escalated in BC over the last decade. This has been, in large part, due to the increasing toxicity of the local drug supply with the mass influx of illicit fentanyl and its analogues (e.g., carfentanyl) [44], as well as the increase in other adulterants including synthetic benzodiazepines such as bromazolam (‘benzo dope’) [9, 60], or sedatives such as xylazine (‘tranqdope’) [9]. In 2023, 43% of unregulated drug overdose deaths^1^in BC involved the use of benzodiazepines, compared to just 3% in 2018 [9]. This change in the drug supply is particularly concerning given that naloxone, while effective in reversing an opioid overdose is, will not reverse the effects of benzodiazepines [8]. Given the rising toxicity of the drug supply, timely response to overdoses (e.g., resuscitation, provision of naloxone and other life-saving measures) is imperative. Thus, a major risk factor for an overdose-related death is using drugs alone as it restricts response time or renders it impossible [20].

Qualitative and survey data have highlighted various social and environmental reasons why people who use drugs (PWUD) use alone, including stigma and fear of discrimination [33, 59, 66, 67], fear of violence [10, 15, 16], not wanting to use in overly populated areas (e.g., shelters) [5], protecting access to limited personal supply [5], wanting privacy [28, 67, 69] lacking trusted peers to use with [80], and having restrictive housing policies (e.g., strict no-guest policies) [40]. Quantitative analyses have shown that individual-level predictors of using drugs alone include binge drug use, preference for using at home, not being in a relationship nor married, and experiencing physical and/or sexual violence [20, 56].

Given the serious risks associated with solitary drug use, there are a number of harm reduction measures in place to address some of the risks people who use alone face, including supervised consumption sites (SCS) and overdose prevention sites (OPS), which are designated locations where trained staff monitor individuals using illicit drugs to prevent or respond to overdoses. However, to maximize the impact of these interventions, SCS can be integrated into housing (e.g., single room occupancy hotels) and shelter settings [15, 16, 46], and expanded to include mobile overdose response services [49, 68]. Additionally, public health messaging in BC has emphasized the dangers of using drugs alone. However, this type of messaging has been critiqued for placing responsibility for overdose risk on the individual, rather than recognizing and addressing the larger contextual and systemic factors influencing this particular drug use practice [7, 43]. Despite the risks and available harm reduction services, between January 1 and October 31, 2024, 81% of people who died from an unregulated drug overdose deaths in BC used alone in private and other residences (e.g., SRO, social and supportive housing) and various other indoor locations (BC [9]). This raises questions regarding how to better address the factors that drive solitary drug use.

Drug use practices and related harms are known to be highly gendered [23, 85]. However, one area that is less researched, and very much overlooked by current public health messaging, is how using drugs alone may be a gendered practice. Previous studies have focused on women in isolation to explore gender-specific social and environmental factors associated with using substances alone (e.g., women’s lived experiences of gender-based violence has been identified as a reason women opt to use alone) [10, 15, 16, 69]. Other studies have found mixed results when considering gender as a variable in quantitative analyses focused on using drugs alone, with one study finding no effect of gender on using alone, and the other finding that men had a higher risk of using alone [20, 56]. This study, however, offers a novel look at the practice of using drugs alone by considering solitary drug use among multiple gender identities separately, and identifying key commonalities and differences, thus moving away from traditional “gender-neutral” approaches to research in this area. Given that using drugs alone is associated with increased risk of overdose-related death [50, 81], we sought to explore solitary drug use practices according to gender in a comparative analysis.

## Methods

### Study design

Data were collected from the Vancouver Injection Drug Users Study (VIDUS), an ongoing prospective cohort study that began in 1996. The study has been described previously in greater detail [47, 75, 79]. To summarize, VIDUS participants include adults who have injected unregulated drugs within the month prior to their enrolment and are HIV-negative. Participants were recruited using community-based methods (e.g., street outreach, snowball sampling, and self-referral) in the Downtown Eastside (DTES) neighbourhood of Vancouver, a neighbourhood in Vancouver which is characterized by high rates of illicit drug use [35]. After written informed consent is obtained, participants receive a $50 honorarium at each study visit where they complete biological sample collection and an interviewer-administered questionnaire, which gathers sociodemographic data as well as data on participants’ drug use behaviours. The study has received ethical approval from the Simon Fraser University Research Ethics Board.

### Study sample and measures

The sample included participants who completed at least one follow-up study between June 2019 and May 2023. From mid-March to mid-July 2020, interviews were suspended due to the COVID-19 pandemic. Between mid-July 2020 to March 2022, interviews were exclusively conducted over the phone, and in-person interviews were resumed in March 2022.

The main explanatory variable, was ascertained by the survey question “In the last six months, how often do you use drugs (injection and non-injection) alone with nobody around (excluding cannabis)?” Possible responses were presented on a Likert scale: always (100% of the time), usually (75% of the time or more), sometimes (26% to 74% of the time), occasionally (25% of the time or less), or never (0% of the time). We created a dichotomous variable (yes vs. no) for solitary drug use (injection or non-injection), where participants who reported using alone “usually” or “always” were coded as “yes” and those who “never”, “occasionally” or “sometimes” used drugs alone were coded as “no”.

Potential correlates were identified a priori based on past studies that assessed using substances alone [25, 56], and our longstanding observational experience in the local environment. Sociodemographic characteristics included: age (per year older); being white (white vs. Black, Indigenous and People of Colour [BIPOC]), relationship status (legally married/common law/regular partners vs. others); and self-identified gender (cisgender men, cisgender women, transgender women, Two-Spirit people, gender diverse people).^2^"Two-Spirit" is a broad umbrella term that encompasses various cultural, spiritual, sexual, and gender identities, with its meaning differing among Indigenous individuals and Nations [77]. For this study, “gender diverse people” includes all participants who did not identify as cisgender, transgender or Two-Spirit, such as participants who identified as “nonbinary” or “other.” The questionnaire asked participants: “In terms of gender identity, how would you best describe yourself?” The question was asked at each follow-up, responses were not prompted (i.e., response options were not provided), and individual responses were coded into the categories listed above after by the interviewer. Participants were able to change their answer at any visit (i.e., participants could self-identify with a different gender at any follow-up). While changes in gender identity have not been commonly reported in cohort studies involving PWUD, our study reveals how the use of emerging best practices in characterizing gender over time avoids obscuring changes in self-reported gender identity. Previous literature has shown that PWUD who identify as a gender minority have distinct lived experiences compared to cisgender PWUD, given that the needs of PWUD are influenced by intersecting social locations (e.g., race, socioeconomic status, gender, sexual orientation). For example, cisgender women and gender diverse PWUD experience unique risks associated with their substance use, including higher exposure to physical and sexual violence, compared to cisgender men [10, 53]. However, we acknowledge that the many genders included within the “gender diverse” category may have distinct experiences of their own, but in order to include these individuals in the analyses we elected to create a broader category due to the small sample size.

During study follow-up, 26 participants reported a new self-identified gender identity (e.g., from “cisgender man” to “transgender woman”); 24 participants reported a different gender identity at a follow-up visit compared to their reported gender at their initial visit, and 2 participants reported different gender identities at 2 follow-up appointments. Accordingly, gender was treated as a time-updated variable, ensuring their data were associated with their self-identified gender at each study follow-up. This serves to recognize the temporal complexities of gender identity (e.g., how changes in gender identity and presentation over time may impact drug use practices) and that to remove these participants or categorize them exclusively into one gender would obscure their unique gendered experiences. This methodology impacted 150 visits in the analysis. Given this, the sum of unique participants in each gender category exceeds the total number of participants in the sample.

Additional factors were measured in the previous six months at each interview. For variables referring to experiences in the past six months, we defined any experience in the past six months (regardless of duration of the experience) as “yes” and no experience in the past six months as “no,” unless otherwise stated. Substance use-related factors included: daily use of any illicit stimulant, including cocaine, crack cocaine, crystal methamphetamine, and MDMA (yes vs. no); daily use of any illicit opioid, including unregulated heroin, fentanyl and down (yes vs. no); binge drug/alcohol use, defined as periods when drugs or alcohol were used more than was usual (yes vs. no). Other characteristics measured in the previous six months included: DTES residence (yes vs. no); homelessness, defined as not having any shelter (yes vs. no); recently experiencing physical and/or sexual violence (yes vs. no); and recent incarceration (yes vs. no) and depression symptomology, measured using the Patient-Reported Outcomes Measurement Information System (PROMIS) short form (8b) Depression Scale (none/mild vs. moderate/severe). We converted raw score to T-scores, with scores less than 60 to indicate “none to mild depressive symptoms”, and scores greater than or equal to 60 to indicate “moderate to severe depressive symptoms” [36].

### Statistical analyses

To examine the characteristics of variables stratified by self-identified gender at baseline, we first estimated crude odds ratios and 95% confidence intervals using a logistic regression analysis for numerical variables. For categorical variables, odds ratios and 95% confidence intervals were calculated using unconditional maximum likelihood estimation and normal approximation, respectively. *P*-values were generated using the Kruskal–Wallis test for numerical variables and Chi-square test for categorical variables.

To determine factors associated with using drugs alone in the previous six months, we conducted an initial bivariate generalized estimating equations (GEE) analysis with a logit link function and an exchangeable correlation structure. Subsequently, we performed a multivariable GEE analysis, incorporating only variables that were significant at the *p* < 0.05 level in the bivariate analyses. We utilized the variance inflation factor (VIF) to examine the existence of multicollinearity (VIF > 5), and found little to no indication of multicollinearity among the variables considered. We conducted analyses involving the entire sample, as well as analyses stratified by self-identified gender, which were conducted using R (version 4.4.1; R Foundation for Statistical Computing, Vienna, Austria), with all *p*-values being two-sided.

## Results

In total 697 individuals participated in this study, including 384 (55.1%) cisgender men, 295 (42.3%) cisgender women, 10 (1.4%) transgender women, 8 (1.1%) Two-Spirit people, 27 (3.9%) gender diverse people (Table 1).^3^ As shown in Table 2, the median age of participants at baseline was 48 years (IQR: 38 – 57), with median age for cisgender men at 52 years (IQR: 26 – 78), 43 years for cisgender women (IQR: 36 – 54), 39 for transgender women (IQR: 35 – 46), 44 years for Two-Spirit people (IQR: 37–48), and 44 for gender diverse people (IQR: 35 – 55). The majority (57.0%) of participants were white, 36.2% were Indigenous, and 6.7% being other people of colour.

**Table 1:** Socio-demographic factors at baseline stratified by gender

Characteristic	All genders	Cisgender Men	Cisgender Women	Transgender Women	Two-Spirit People	Gender Diverse People^~^	
	**n (%) *n* = 697**^**a**^	**n (%) *n* = 384**^**a**^	**n (%) *n* = 295**^**a**^	**n (%) *n* = 10**	**n (%) *n* = 8**	**n (%) *n* = 27**	
**Age** median (IQR)	48 (38–57)	52 (40–59)	43 (36–54)	39 (35–46)	44 (37–48)	44 (35–55)	
**white** (white vs. BIPOC^c^)	397 (57.0)	259 (67.4)	129 (43.7)	7 (70.0)	1 (12.5)	14 (51.9)	
**Homeless**^**d**^ (yes vs. no)	130/688 (18.9)	74/379 (19.5)	53/291 (18.2)	2 (20.0)	1 (12.5)	4 (14.8)	
**Daily Illicit Stimulant Use**^**d**^ (yes vs. no)	262/696 (37.6)	115/383 (30.0)	138 (46.8)	3 (30.0)	5 (62.5)	11 (40.7)	
**Daily Illicit Opioid Use**^**d**^ (yes vs. no)	295/696 (42.4)	144/383 (37.6)	143 (48.5)	4 (40.0)	4 (50.0)	13 (48.1)	
**Binge Use**^**d,e**^ (yes vs. no)	184**/**586 (31.4)	97/331 (29.3)	78/237 (32.9)	3/6 (50.0)	2/3 (66.7)	6/15 (40.0)	
**Relationship**^**f**^ (yes vs. no)	214**/**640 (33.4)	88/354 (24.9)	120/269 (44.6)	2 (20.0)	1 (12.5)	10/26 (38.5)	
**Recent Incarceration**^**d**^ (yes vs. no)	42/694 (6.1)	28 (7.3)	14/292 (4.8)	0 (0.0)	0 (0.0)	0 (0.0)	
**Victim of Violence**^**d**^ (yes vs. no)	98/689 (14.2)	50/382 (13.1)	43/289 (14.9)	3 (30.0)	1 (12.5)	5 (18.5)	
**DTES Residence**^**d,g**^ (yes vs. no)	452 (64.8)	239 (62.2)	200 (67.8)	8 (80.0)	6 (75.0)	6 (75.0)	
**Depression**^**h**^ (moderate/severe vs. none/mild)	189/633 (29.9)	76/352 (21.6)	104/266 (39.1)	4/9 (44.4)	5/7 (71.4)	9/24 (37.5)	

^a^Some questions were not answered by participants; percentages reported are based the subsample without missing values to give a better estimate of population prevalence

^b^CI: confidence intervals

^c^BIPOC: Black, Indigenous and People of Colour

^d^Experienced within the previous 6 months

^e^Binge use: periods when drugs or alcohol were used more than usual

^f^Relationship: being legally married, having a common law or regular partner

^g^DTES: Downtown Eastside, Vancouver

^h^Measured using PROMIS Depression Scale. T-scores < 60 = “none/mile,” and scores ≥ 60 = “moderate/severe.”

^~^Anyone who did not identify as cisgender, transgender or Two-Spirit (e.g., nonbinary)

**Table 2:** Socio-demographic factors at baseline, entire sample stratified by solitary drug use in last 6 months (*n* = 697)

Characteristic	Total sample *n* = 697^a^	Solitary use n (42.6%) *n* = 297^a^	No solitary use n (57.4%) *n* = 400	*p –* value^^^
**Age** median (IQR)	48 (38 – 57)	48 (38 – 58)	48 (38 – 57)	0.447^*^
**white** (white vs. BIPOC^c^)	397 (57.0)	183 (61.6)	214 (53.5)	0.037
**Homeless**^**d**^ (yes vs. no)	130/688 (18.9)	54/294 (18.4)	76/394 (19.3)	0.769
**Daily Illicit Stimulant Use**^**d**^ (yes vs. no)	262/696 (37.6)	140/296 (47.3)	122/400 (30.5)	< 0.001
**Daily Illicit Opioid Use**^**d**^ (yes vs. no)	295/696 (42.4)	144/296 (48.6)	151/400 (37.8)	0.004
**Binge Use**^**d,e**^ (yes vs. no)	184/586 (31.4)	102/262 (38.9)	82/324 (25.3)	< 0.001
**Relationship**^**f**^ (yes vs. no)	214/640 (33.4)	60/280 (21.4)	154/360 (42.8)	< 0.001
**Recent Incarceration**^**d**^ (yes vs. no)	42/694 (6.1)	15/296 (5.1)	27/398 (6.8)	0.422
**Victim of Violence**^**d**^ (yes vs. no)	98/689 (14.2)	53/294 (18.0)	45/395 (11.4)	0.015
**DTES Residence**^**d,g**^ (yes vs. no)	452 (64.8)	210 (70.7)	242 (60.5)	0.006
**Depression**^**h**^ (moderate/severe vs. none/mild)	189/633 (29.9)	103/275 (37.5)	86/358 (24.0)	< 0.001

^a^ Some questions were not answered by participants; percentages reported are based the subsample without missing values to give a better estimate of population prevalence

^b^ CI: confidence intervals. population prevalence

^c^ BIPOC: Black, Indigenous and People of Colour

^d^ Experienced within the previous 6 months

^e^ Binge use: periods when drugs or alcohol were used more than usual

^f^ Relationship: being legally married, having a common law or regular partner

^g^ DTES: Downtown Eastside, Vancouver

^h^ Measured using PROMIS Depression Scale. T-scores < 60 = “none/mile,” and scores ≥ 60 = “moderate/severe.”

^^^ Fischer’s test ^*^ Kruskal–Wallis test

Two-Spirit people and cisgender women were more likely than to use both illicit stimulants (total: 37.6%; cisgender men: 30.2%; cisgender women: 46.8%; transgender women: 30.0%; Two-Spirit people: 62.5%; gender diverse people: 40.7%). Transgender women, Two-Spirit and gender diverse people engaged more in binge drug use (total: 31.4%; cisgender men: 29.3%; cisgender women: 32.9%; transgender women: 50.0%; Two-Spirit people: 66.7%; gender diverse people: 40.0%). Transgender women, Two-Spirit people and gender diverse people engaged more in binge drug use (total: 31.4%; cisgender men: 29.3%; cisgender women: 32.9%; transgender women: 50.0%; Two-Spirit people: 66.7%; gender diverse people: 40.0%). A higher proportion of cisgender women and gender diverse people were in relationships compared to other groups (total: 33.4%; cisgender men: 24.9%; cisgender women: 44.6%; transgender women: 20%%; Two-Spirit people: 12.5%; gender diverse people: 38.5%). Two Spirit people were much more likely to have depression than others, followed by transgender women, cisgender women and gender diverse people (total: 29.9%; cisgender men: 21.6%; cisgender women: 39.1%; transgender women: 44.4%; Two-Spirit people: 71.4%; gender diverse people: 37.5%).

The median number of follow-up questionnaires each participant completed was 5 (IQR: 1 – 8), and we observed 3144 follow-ups in total. There were 297 (42.6%) participants reported using drugs alone in the previous 6 months at baseline: 44.5% of cisgender men, 39.3% of cisgender women, 30% of transgender women, 25% of Two-spirit people and 48.1% of gender diverse people. In total, 452 (64.8%) participants reported using alone at least once during study follow-ups: 65.6% of cisgender men; 63.1% of cisgender women; 80.0% of transgender women; 87.5% of Two-Spirit people; 77.8% of gender diverse people). The bivariate and multivariable GEE analysis results are shown in Tables 3 and 4, respectively, and show results for the unstratified analysis (‘combined analysis’) and gender-stratified analyses.

**Table 3:** Bivariate GEE^a^ analyses of factors associated with using drugs alone in the last 6 months. Unadjusted odds ratio for regression analyses, with *p*-values

Characteristic	All genders^^^		Cisgender Men		Cisgender Women		Transgender Women		Two-Spirit People		Gender Diverse People^~^	
	**Odds Ratio (95% CI**^**b**^**)**	***p –* value**	**Odds Ratio (95% CI**^**b**^**)**	***p –* value**	**Odds Ratio (95% CI**^**b**^**)**	***p –* value**	**Odds Ratio (95% CI**^**b**^**)**	***p –* value**	**Odds Ratio (95% CI**^**b**^**)**	***p –* value**	**Odds Ratio (95% CI**^**b**^**)**	***p –* value**
**Age** (median)	0.99 (0.99–1.00)	0.119	0.99 (0.98–1.01)	0.399	0.99 (0.97–1.01)	0.351	1.07 (0.93–1.23)	0.336	0.94 (0.85–1.04)	0.227	1.04 (0.98–1.09)	0.204
**Gender** (cisgender men vs. not cisgender men identifying)	**0.83** (0.72 – 0.97)	**0.015**	**–**	**–**	**–**	**–**	**–**	**–**	**–**	**–**	**–**	**–**
**white** (white vs. BIPOC^c^)	**1.30** (1.12 –1.51)	** < 0.001**	**1.59** (1.11–2.27)	**0.011**	0.94 (0.63–1.38)	0.663	**–**	**–**	**–**	**–**	2.13 (0.80–5.71)	0.131
**Homeless**^**d**^ (yes vs. no)	0.95 (0.77–1.16)	0.596	**0.65** (0.47–0.91)	**0.013**	1.01 (0.71–1.43)	0.973	**–**	**–**	**–**	**–**	1.44 (0.35–5.94)	0.612
**Daily Illicit Stimulant Use**^**d**^ (yes vs. no)	**1.68** (1.45–1.95)	** < 0.001**	**1.78** (1.39–2.29)	** < 0.001**	**1.41** (1.09–1.84)	**0.010**	0.56 (0.13–2.52)	0.452	1.00 (0.16–6.35)	1.000	1.98 (0.61–6.39)	0.254
**Daily Illicit Opioid Use**^**d**^ (yes vs. no)	**1.92** (1.66–2.23)	** < 0.001**	**1.59** (1.22–2.07)	** < 0.001**	**1.39** (1.06–1.82)	**0.017**	1.88 (0.42–8.45)	0.410	**-**	-	**3.17** (1.10–9.13)	**0.033**
**Binge Use**^**d,e**^ (yes vs. no)	**1.76** (1.32–2.36)	** < 0.001**	**2.05** (1.38–3.04)	** < 0.001**	**1.76** (1.07–2.92)	**0.027**	–	–	–	–	2.50 (0.29–21.41)	0.403
**Relationship**^**f**^ (yes vs. no)	**0.33** (0.27–0.40)	** < 0.001**	**0.46** (0.33–0.64)	** < 0.001**	**0.54** (0.39–0.74)	** < 0.001**	1.49 (0.19–11.37)	0.703	-	-	0.53 (0.15–1.87)	0.323
**Recent Incarceration**^**d**^ (yes vs. no)	0.97 (0.64–1.45)	0.874	1.14 (0.72–1.81)	0.579	0.93 (0.59–1.48)	0.774	**–**	**–**	**–**	**–**	**–**	**–**
**Victim of Violence**^**d**^ (yes vs. no)	**1.49** (1.22–1.81)	** < 0.001**	1.08 (0.82–1.43)	0.588	1.00 (0.75–1.34)	0.986	2.27 (0.50–10.32)	0.288	0.29 (0.02–3.60)	0.336	**9.63** (1.11–83.50)	**0.040**
**DTES Residence**^**d,g**^ (yes vs. no)	**1.49** (1.28–1.73)	** < 0.001**	1.13 (0.86–1.48)	0.392	**1.46** (1.05–2.03)	**0.026**	**0.18** (0.03–0.97)	**0.046**	1.50 (0.27–8.36)	0.644	**2.61** (1.05–6.44)	**0.038**
**Depression**^**h**^ (moderate/severe vs. none/mild)	**1.65** (1.40–1.95)	** < 0.001**	**1.33** (1.04–1.70)	**0.023**	**1.43** (1.07–1.92)	**0.016**	1.67 (0.34–8.26)	0.532	**8.00** (1.70–37.67)	**0.009**	**5.05** (1.52–16.78)	**0.008**

^a^GEE: generalized estimating equation

^b^CI: confidence intervals

^c^BIPOC: Black, Indigenous and People of Colour

^d^Experienced within the previous 6 months

^e^Binge use: periods when drugs or alcohol were used more than usual

^f^Relationship: being legally married, having a common law or regular partner

^g^DTES: Downtown Eastside, Vancouver

^h^Measured using PROMIS Depression Scale. T-scores < 60 = “none/mile,” and scores ≥ 60 = “moderate/severe.”

^^^Comparison of men vs women, where “women” is inclusive of transwomen, Two-Spirit people and gender diverse people

^~^Anyone who did not identify as cisgender or transgender

**Table 4:** Multivariable GEE^a^ analyses of factors associated with using drugs alone in the last 6 months. Adjusted odds ratio (AOR) for regression analyses, with *p*-values

Characteristic	All genders^^^		Cisgender Men		Cisgender Women	
	**AOR (95% CI**^**b**^**)**	***p –* value**	**AOR (95% CI**^**b**^**)**	***p –* value**	**AOR (95% CI**^**b**^**)**	***p –* value**
**Age** (median)	**-**	**-**	**-**	***-***	**-**	***-***
**Gender** (cisgender men vs. not cisgender men identifying)	0.70 (0.49 – 1.00)	0.050	**-**	**-**	**-**	***-***
**white** (white vs. BIPOC^c^)	1.22 (0.86 – 1.73)	0.262	1.05 (0.64 – 0.71)	0.848	**-**	***-***
**Homeless**^**d**^ (yes vs. no)	-	-	**0.45** (0.25 – 0.82)	**0.010**	**-**	***-***
**Daily Illicit Stimulant Use**^**d**^ (yes vs. no)	**1.75** (1.22 – 2.50)	**0.002**	**1.90** (1.17 – 3.10)	**0.010**	1.63 (0.93 – 2.86)	0.086
**Daily Illicit Opioid Use**^**d**^ (yes vs. no)	1.38 (0.97 – 1.97)	0.072	1.34 (0.83 – 2.15)	0.227	1.65 (0.91 – 3.00)	0.102
**Binge Use**^**d,e**^ (yes vs. no)	**1.70** (1.21 – 2.37)	**0.002**	**2.18** (1.38 – 3.45)	** < 0.001**	1.41 (0.79 – 2.53)	0.245
**Relationship**^**f**^ (yes vs. no)	**0.30** (0.21 – 0.44)	** < 0.001**	**0.25** (0.14 – 0.43)	** < 0.001**	**0.34** (0.19 – 0.62)	** < 0.001**
**Recent Incarceration**^**d**^ (yes vs. no)	**-**	**-**	**-**	***-***	**-**	***-***
**Victim of Violence**^**d**^ (yes vs. no)	1.07 (0.66–1.75)	0.779	**-**	***-***	**-**	**-**
**DTES Residence**^**d,g**^ (yes vs. no)	1.02 (0.71 – 1.46)	0.922	**-**	***-***	0.87 (0.47 – 1.62)	0.655
**Depression**^**h**^ (moderate/severe vs. none/mild)	**1.73** (1.19 – 2.51)	**0.004**	1.42 (0.85 – 2.37)	0.181	**2.16** (1.20 – 3.89)	**0.011**

^a^GEE: generalized estimating equation

^b^CI: confidence intervals

^c^BIPOC: Black, Indigenous and People of Colour

^d^Experienced within the previous 6 months

^e^Binge use: periods when drugs or alcohol were used more than usual

^f^Relationship: being legally married, having a common law or regular partner

^g^DTES: Downtown Eastside, Vancouver

^h^Depression was measured using the PROMIS short form (8b) Depression Scale. T-scores < 60 = “none/mile,” and scores ≥ 60 = “moderate/severe.”

^^^ Comparison of men vs women, where “men” is inclusive of transmen (note that there are none in the sample) and “women” is inclusive of transwomen, Two-Spirit individuals and all other non-binary genders

### Bivariate GEE analyses

The results from the bivariate combined analyses (Table 3) show 8 statistically significant factors that impact the likelihood of using alone, while the gender stratified analyses found 6 statistically significant factors impacting the odds of using alone for cisgender men, 3 factors for cisgender women, one factor for transgender women and Two-Spirit people, and 4 factors for gender diverse people. Of note, the bivariate analyses indicated a larger impact of depression on using alone for Two-Spirit (OR = 8.00, %95CI: 1.70 – 37.67) and gender diverse people (OR = 5.05, 95%CI: 1.52 – 16.78) compared to cisgender men (OR = 1.65, 95%CI: 1.04 – 1.70) and cisgender women (OR = 1.43, 95%CI: 1.07 – 1.92), and only gender diverse people’ risk was impacted by experiences of violence (OR = 9.63, 95%CI: 1.11 – 83.50) compared to all other genders. All *p*-values < 0.05.

Given the small sample size of transgender women, Two-Spirit people and gender diverse people, we were only able to move forward with multivariable analyses for cisgender men, cisgender women and a combined analysis of all genders (inclusive of all 5 gender categories).

### Multivariable (adjusted) GEE analyses

In the multivariable combined analysis (Table 4), the results reveal 6 statistically significant factors that impact the likelihood of participants using alone. Factors that increase the likelihood of solitary drug use include using illicit stimulants daily (AOR = 1.74, 95%CI: 1.22 – 2.46, *p* = 0.002), using illicit opioids daily (AOR = 1.37, 95%CI: 0.97 – 1.95, *p* = 0.076), binge use of drugs (AOR = 1.69, 95%CI: 1.21 – 2.36), and having moderate to severe depression (AOR = 1.75, 95%CI: 1.21 – 2.54, *p* = 0.003). Factors that decrease the odds of using drugs alone include being in a relationship (AOR = 0.31, 95%CI: 0.21 – 0.45, *p* < 0.001).

In gender-stratified multivariable GEE analyses, four statistically significant factors impacted the odds of using drugs alone for cisgender men and two factors for cisgender women. Being in a relationship was negatively associated for both cisgender men and women (cisgender men: AOR = 0.25, 95%CI: 0.15 – 0.44, *p* < 0.001; cisgender women: AOR = 0.37, 95%CI: 0.22 – 0.63, *p* < 0.001), while homelessness was negatively associated with cisgender men only (AOR = 0.50, 95%CI: 0.27 – 0.91, *p* = 0.015). Factors increased the likelihood for cisgender men included daily illicit stimulant use (AOR = 1.83, 95%CI: 1.13 – 2.96, *p*. = 0.005), and binge drug use (AOR = 2.21**,** 95%CI: 1.40 – 3.48, 0.001). For cisgender women, factors positively associated included only binge use (AOR = 1.88, 95%CI: 1.10 – 3.23, *p* = 0.022).

## Discussion

While there is some evidence that solitary drug use does not differ by gender [59], the findings from our gender-stratified analyses suggest that the practice of solitary illicit drug use remains prevalent and is gendered, with unique factors shaping the practice among cisgender men and women. Our study aligns with previous research indicating that being in a relationship is a protective factor against using drugs alone [20, 56, 65], a factor that has been found to function as a form of social protection and mutual care in reducing drug-related harms [65]. This is salient given that many residences that participants in this study reside in (e.g., SROs) have restrictive guest policies (e.g., prohibiting overnight guests, restricting non-resident guests to common spaces) [40], which may hinder someone from spending time with their partner and using drugs with them. However, there is evidence that using drugs with intimate partners may negatively impact women in relationships with men given the increased risk of experiencing intimate partner violence (IPV) [31], and the decreased ability to negotiate safer consumption practices (e.g., using a previously used syringe) [70]. Thus, while we found that being in a relationship decreases the odds of using alone, we cannot be certain that this is a positive condition for both men and women, as there may be additional concerns relating to this issue that are unseen in our analyses.

The positive relationship between binge use and the likelihood of using alone has been noted within another quantitative study of PWUD in the DTES [56]. That particular study’s methodology included both men and women in a single analysis, which may have hidden the gendered impact of binge drug use. Indeed, the findings from our combined analysis also demonstrate that binge drug use increases the odds of using alone, but when we conducted two separate analyses stratified by gender, we see that binge use only increases the odds for cisgender men – not for cisgender women.

Homelessness and housing instability has been identified by others as increasing the risk of using alone and in hidden public areas (e.g., alleyways, parks) [29, 59]. Other have found that obtaining secure independent housing was associated with increased solitary drug use among people who had previously experienced homelessness [83]. This may be due to social norms that encourage group drug use in certain types of housing (e.g., single-room occupancy hotels), which decrease the likelihood of using alone [83]. Housing precarity also has other impacts on high-risk drug use patterns (e.g., increased risk for syringe sharing) [37] in general, and for specific populations, the initiation of injection drug use among youth as well [24]. While homelessness was not found to impact odds of using alone in our combined analyses, in our gender-stratified analyses, homelessness was protective against using drugs alone among men but not women. Future research should seek to unpack this association, with attention to gendered aspects of homelessness.

Our findings show that the types of substances used had differential impacts on cisgender men’s risk of solitary drug use, with daily illicit stimulant use increasing their risk, but not daily illicit opioid use. Neither type of daily drug use impacted cisgender women’s risk. While men have found to be more likely to use have a stimulant use disorder than woman [51], there has not been a greater investigation into the motivating factors behind this difference. Our finding, though less interpretable, highlights the importance of stratifying analyses to better understand how substance use patterns vary across different groups. For instance, in our unstratified analysis, daily stimulant use is a significant factor increasing the likelihood of using drugs alone, but when stratified by gender, this significance is observed only in cisgender men.

A finding unique to our study is the increased odds of using alone among cisgender women with moderate to severe depression. This finding is inconsistent with a previous study that found no association between solitary drug use and depression [56]; however, the study did not stratify its analysis by gender, which may have obscured gender-specific dynamics. The association between depression and social isolation is well-established, however, it has not been definitively determined whether social isolation leads to depression [42], if depression leads to social isolation [48], or if there is a bidirectional relationship [21, 26, 71]. Among PWUD, this relationship suggests that depression may be both a cause and consequence of solitary drug use, whereby solitary drug use – motivated by any number of factors such as wanting privacy, ensuring safety, convenience, etc. – may lead to depression, and in turn depression may increase solitary drug use. Past research involving PWUD in Vancouver has found that women tend to report higher levels of depression than men, and this has been associated with elevated risk of, violence [76] and non-fatal overdose [58]. Our findings suggest even greater urgency in providing effective mental health care and social supports to women who use drugs.

However, while efforts are needed to address depression and associated risks among women who use drugs, recent evidence raises concerns about conventional pharmacotherapy for depression. Aside from emerging questions regarding the benefits of modern antidepressant medications [41, 55], a growing body of evidence suggests that these medications, in particular serotonergic antidepressants, are ineffective in substance using populations, and in some cases may exacerbate cravings and intensity of substance use [1, 18, 32, 38, 73, 74, 86]. However, other pharmacotherapies and certain psychological interventions substantially improve outcomes among those engaged in high-risk substance use, and should be explored where appropriate [12, 45, 82].

### Implications for targeted interventions and policies

As previously stated, public health messaging targeted at people who use drugs alone in BC has focused on the increased risk of having a fatal overdose, and emphasized the importance of using drugs with other people. However, people continue to use drugs alone; indeed, 78% of participants reported solitary drug use at least once throughout this study alone. However, informal community driven interventions have long been in place to support PWUD, among them is “spotting” – letting others know when you plan to taking drugs alone to have a witness or someone to check in on you to increase overdose response [62]. This practice has been noted as an important risk mitigation strategy for women who find increased safety in using alone given their past experiences and fear of physical harm [69]. The current public health discourse of discouraging solitary drug use does not fulsomely take into account people’s wide range of reasons and risk factors for using alone. Instead, recognizing and encouraging other ways to ‘stay safe’ while using alone (e.g., spotting), affirms that using drugs alone may be a safety plan in itself, while identifying other ways to increase overdose response time.

One way in which we can support people who use drugs alone to remain safer is by using technologies inspired by “spotting.” Digital harm reduction services via mobile apps have emerged in the last decade, targeted toward connecting PWUD who would otherwise use drugs alone to others who can supervise them remotely and provide overdose support if necessary [22, 78]. One such technology used in the DTES is the Brave Button, which is located throughout some supportive housing buildings in the neighbourhood. When pressed, the button alerts building staff via text that a resident is about to use drugs (presumably alone) allowing staff to check in on the resident to ensure their safety [14]. These mobile solutions have the potential to prevent overdose-related deaths, however, their effectiveness hinges on securing the privacy of app users and trust between PWUD and provider [22, 78]. However, mobile solutions do have drawbacks that are specific to this population. For example, these apps may not accessible for those without consistent access to cell phone and/or internet, and may not be suitable for people experiencing homelessness or frequently changing living environments [78].

While there are SCS in Vancouver, women have identified many barriers to accessing these SCS, as well as describing them as potential sites of gendered and racialized violence [4, 10]. Women-only (transgender, Two-Spirit and nonbinary inclusive) SCS have been identified as an important intervention to reduce solitary drug use among women. However, the programming and physical environment of these programs is important for reducing barriers to access. Considerations such as ensuring privacy [2], promoting diversity (gender diverse inclusive) [2, 11], extended hours [84], and providing basic needs (e.g., food) [2, 11], and a non-institutional and de-medicalized setting [2, 11] can improve access to these sites.

When discussing risk and driving factors associated with solitary drug use, it’s critical to recognize and affirm that PWUD’s choices regarding high-risk drug use practices are constrained by drug policies (e.g., criminalization) and structural forces (e.g., access to low-barrier housing), which counters the idea that PWUD are entirely responsible for their decisions and the potential resulting harms [30, 64]. Recommendations that focus solely on addressing individual-level concerns without addressing larger systemic barriers will likely have limited impact in the long run. Thus, there is a need to look to interventions and policies that address these broader issues and are gender-responsive.

Given that over 75% of all overdose-related deaths occur in indoor residences in BC [9], there is substantial opportunity to address this issue through housing-based harm reduction measures. In particular, integrating OPS into housing frequently accessed by PWUD (including SROs and shelters) to easily accessible and more secure locations have the potential to reduce drug-related harms [6, 46]. Greater physical accessibility to supervised consumption via housing-based OPS or “HOPS” may reduce incidence of using drugs alone, given wait times and general inconvenience cited as barriers to access [13, 59].

However, simply implementing HOPS may not be sufficient for some populations. For example, women who use drugs in the BC have reported feeling unsafe and experiencing racialized and gendered violence in both OPS and within HOPS in their residence [10, 15, 16]. Indeed, fear of violence has been identified as a unique barrier to accessing harm reduction services for women [34]. Women-only OPS have been noted as creating safer spaces for women [4, 72], and implementing women-only HOPS (in all genders as well as women-only housing) may increase supervised drug use among women who use drugs.

Another population to consider are those who predominantly consume drugs via inhalation. In our study, 469 individuals (67.3%) reported non-injection drug use (including inhalation as a method of consumption) in the last 6 months prior to their visit. Inhalation is fast becoming the predominant method of consumption of drugs in BC [61], with 68% of unregulated drug overdose deaths in 2024 (January 1–September 30) resulting from smoking as a mode of drug consumption [9]. A 2019 study in BC demonstrated that smoking opioids was significantly associated with solitary drug use, with people who used drugs alone being nearly 3 times more likely to smoke opioids than those who did not [61]. Non-injection drug use has been marginalized within harm reduction services, which are typically oriented toward reducing the health risks associated with injection drug use. Despite the evidence that people who smoke have high willingness to use supervised smoking facilities (SSF) [17, 19], their implementation and uptake has been slow. Increasing supervised spaces where people can smoke has the potential to reduce unsupervised substance use.

Safe supply programs, which distribute a legal and regulated supply of designated drugs, have been shown to decrease overdoses and overdose-related deaths, as well as reducing PWUD’s use of the toxic, unregulated drug supply [27, 39, 40]. Criminalization of drugs promotes stigma against PWUD [3], which is a well-cited reason for using drugs alone [33, 59, 66, 67]. By regulating drugs and providing access to a safe supply, the agency of PWUD is supported and interactions with the criminal law system can be reduced [57].

### Limitations

This study has limitations to note. As previously mentioned, 26 participants reported a new self-identified gender identity at a follow-up questionnaire. While we stand by the decision to treat gender as a time-updated variable, it resulted in the sum of unique participants in each gender category exceeding the total number of participants in the sample. Additionally, the small sample sizes of transgender women, Two-Spirit, and gender diverse people limited our analyses to bivariate GEE models for these groups. We recommend future research that investigates the unique solitary drug use patterns of gender minorities in more detail. Another limitation to declare is that the participants in this study were recruited in the DTES of Vancouver, using a non-random sample (recruited via community-based methods), which may limit generalizability of findings to all PWUD in Vancouver. In addition, data was self-reported, which is subject to reporting biases such as socially desirable responding and recall bias. Additionally, we did not ask participants about overdose prevention measures that they may have taken when using alone (e.g., spotting, etc.). So, our outcome measure (using alone) does not immediately mean a risk behaviour for fatal overdose.

## Conclusion

The findings of this study suggest significant heterogeneity in gender-specific factors associated with using drugs alone. Substance use differentially affected the risk of using drugs alone according to gender, with depression having a significant impact on cisgender women’s, but not cisgender men’s, risk. Additionally, the risk of using drugs alone for cisgender men and cisgender women varied based on daily use of specific drugs, with cisgender men’s risk increased by daily illicit stimulant use. While unable to conduct a multivariable analysis on transgender, Two-Spirit and gender diverse people due to small sample sizes, bivariate analyses showed larger impact of depression on using alone for Two-Spirit and gender diverse people compared to other gender identities, and only gender diverse people’s risk was impacted by experiences of violence.

There are a multitude of reasons and risk factors for why people use drugs alone. When we shift the narrative away from condemning solitary drug use and toward addressing the issues preventing PWUD from using with others and/or finding other ways to reduce harms while using alone, we could improve conditions for PWUD. Ultimately, gender-specific factors must be recognized in public health messaging, and in developing policies and harm reduction measures to address the risks associated with using alone.

## Data Availability

The dataset from the Vancouver Injection Drug User Study (VIDUS) is not publicly available due to ethics requirements, but may be available from the corresponding author on reasonable request.

## Abbreviations

BC: British Columbia
DTES: Downtown Eastside, Vancouver
GEE: Generalized estimating equations
HOPS: Housing-based overdose prevention site
IPV: Intimate partner violence
OPS: Overdose prevention site
PROMIS: Patient Reported Outcomes Measurement Information System
PWUD: People who use drugs
SCS: Supervised consumption site
SRO: Single-room occupancy (housing unit)
SSF: Supervised Smoking Facility
VIDUS: Vancouver Injection Drug Use Study

## Footnotes

1 Accidental or undetermined drug poisoning deaths from use of unregulated drugs and/or controlled drugs sold illicitly.

2 There were no individuals who identified as a transgender man in our study sample.

3 This does not add to 697 participants given that 26 individuals changed their self-identified gender during study follow-up. Their genders were retrospectively contributed to more than one baseline table.
